# NLRP3 Inflammasome Activation‐Induced Acute Papillitis as a Trigger of Acute Pancreatitis ‐ A Novel Mechanism of Microlithiasis‐Induced Acute Pancreatitis

**DOI:** 10.1002/ueg2.70198

**Published:** 2026-03-14

**Authors:** Simon Sirtl, Mahmood Ahmad, Prince Allawadhi, Thomas Metzler, Oliver Buchstab, Steffen Ormanns, Martina Rudelius, Georg Beyer, Lukasz Krupa, Robert Staron, Christian Schulz, Lisa Fahr, Andrea Sendelhofert, Christian Schulz, Katja Steiger, Matthias Sendler, Markus M. Lerch, Ivonne Regel, Michal Żorniak, Julia Mayerle, Ujjwal M. Mahajan

**Affiliations:** ^1^ Department of Medicine II University Hospital LMU Munich Germany; ^2^ Comparative Experimental Pathology (CEP) Technical University of Munich Munich Germany; ^3^ Faculty of Medicine Institute of Pathology LMU Munich Germany; ^4^ Institute of General Pathology Medical University Innsbruck Innsbruck Austria; ^5^ Innpath Institute of Pathology Tirol Kliniken Innsbruck Austria; ^6^ Department of Gastroenterology and Hepatology with Internal Disease Unit Medical Department University of Rzeszów Rzeszów Poland; ^7^ Department of Medicine I University Hospital LMU Munich Germany; ^8^ Department of Immunopharmacology Medical Faculty Mannheim Mannheim Institute for Innate Immunoscience (MI3) Heidelberg University Mannheim Germany; ^9^ Department of Medicine A University Medicine Greifswald Greifswald Germany; ^10^ LMU University Hospital Ludwig Maximilian Universität München Munich Germany; ^11^ Endoscopy Department Maria Sklodowska‐Curie National Research Institute of Oncology Gliwice Poland; ^12^ Department of Pharmacology and Toxicology National Institute of Pharmaceutical Education and Research (NIPER) Mohali India

**Keywords:** acute pancreatitis, biliary pancreatitis, experimental pancreatitis, gallstone, microlithiasis, NLRP3, NLRP3 inflammasome, sludge

## Abstract

**Introduction:**

Obstruction of the pancreatic duct by impacted gallstones at the level of the papilla vateri causes acute pancreatitis. How non‐obstructing stones such as microlithiasis or sludge cause pancreatitis has not been studied. We aimed to understand the pathomechanism of microlithiasis‐induced acute pancreatitis.

**Methods:**

In human papillary biopsies from patients with microlithiasis‐induced acute pancreatitis (*n* = 4), alcohol‐induced acute pancreatitis (*n* = 5), and control subjects without pancreatobiliary disease (*n* = 4), the inflammatory infiltrate was quantified. Bone marrow–derived macrophages generated from C57BL/6 mice were treated in vitro with cholesterol monohydrate and calcium bilirubinate crystals, and NLRP3 inflammasome‐mediated macrophage activation was quantified. Microlithiasis formation in the gallbladder was induced in mice through lithogenic high fat diet and devazepide. Acute pancreatitis was induced by supramaximal caerulein stimulation. Microlithiasis ejection from the gallbladder was achieved through low‐dose caerulein i.p. Injections. Pancreatitis severity was compared between caerulein‐induced pancreatitis and caerulein‐induced pancreatitis after repetitive microlithiasis ejection.

**Results:**

Significantly higher infiltration of CD45‐positive leukocytes and increased NLRP3 expression were observed in papillary biopsies from patients with microlithiasis‐induced acute pancreatitis compared with patients with alcohol‐induced acute pancreatitis and control subjects. In line with this, significantly higher IL‐1ß secretion and caspase‐1 activation were observed in vitro in bone marrow‐derived macrophages stimulated with cholesterol monohydrate and calcium billirubinate crystals. In vivo microlithiasis formation was achieved in all mice with high fat diet and devazepide. Compared to caerulein‐induced pancreatitis, in caerulein + microlithiasis pancreatitis, higher LDH, GPT and ALP levels in serum were observed, but without an impact on pancreatitis severity. However, mice papilla mimicked the phenotype of microlithiasis‐induced acute pancreatitis in humans.

**Conclusion:**

We propose a novel mechanism in which biliary microlithiasis induces a local inflammatory reaction at the papilla (acute papillitis) via NLRP3 inflammasome activation driven by bone marrow‐derived macrophages, without causing pancreatic outflow obstruction.

## Key Summary

- Summarize the established knowledge on this subject
  ◦ Gallstone‐induced obstruction of the pancreatobiliary outflow tract is understood to cause biliary pancreatitis
  ◦ Microlithiasis or sludge is suspected to cause acute pancreatitis, but the pathomechanism is not fully understood. Unlike larger gallstones, pancreatic outflow obstruction is unlikely to be caused by biliary microlithiasis or sludge
- What are the significant and/or new findings of this study?
  ◦ Human papillary biopsies from patients with microlithiasis‐induced acute pancreatitis show an increased inflammatory infiltrate as well as increased NLRP3 expression reflecting a DAMP‐driven state of papillitis
  ◦ Cholesterol crystals as well as bilirubinate crystals as major components of microlithiasis and sludge can induce NLRP3 inflammasome activation in pro‐inflammatory bone‐marrow derived macrophages
  ◦ In a novel mouse model mimicking the proposed mechanism of microlithiasis‐induced pancreatitis we recapitulated papillitis of the papilla vateri

## Graphical abstract

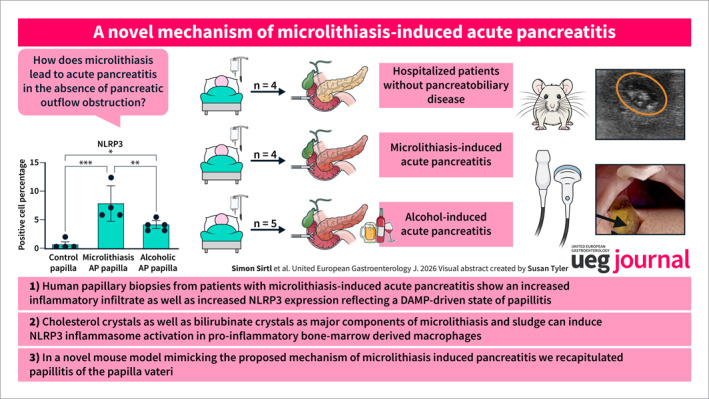

## 1 Introduction

As early as 1901, Eugene L. Opie described gallstone‐related pancreatobiliary outflow obstruction as the key mechanism of biliary acute pancreatitis (AP) [1]. Gallstones migrating from the gallbladder obstruct the level of the major duodenal papilla and consecutively lead to the initiation of the pancreatitis signaling cascade. Animal studies in opossums and mice have shown that acinar cell necrosis and pancreatic edema formation occur after duct blockage [2, 3, 4, 5]. In a recent study, it was shown that sludge and microlithiasis can be detected endosonographically in almost one in five patients with idiopathic AP [6]. However, unlike gallstone‐related pancreatobiliary outflow obstruction, there is a lack of mechanistic understanding of how biliary sludge and microlithiasis, in the absence of mechanical duct obstruction can cause AP.

The lack of appropriate animal models reflecting the complexity of the disease's pathophysiology might explain our inadequate mechanistic understanding [7, 8, 9]. Existing mouse models for biliary AP, such as those involving retrograde infusion of sodium taurocholate or surgical blockage of the pancreatobiliary outflow tract, are considered too artificial to imitate human biliary pancreatitis accurately [10]. In animal models, gallstone‐induced pancreaticobiliary tract obstruction is often simulated by duct ligation, which is considered a critical trigger for pancreatitis [3]. However, the fact that biliary microlithiasis does not result in bile duct obstruction introduces complexity to the understanding of its role in triggering AP. This discrepancy highlights a substantial gap in our understanding of the disease mechanism and underscores the need for further studies into the pathways through which microlithiasis and sludge contribute to AP.

The NLRP3 inflammasome has been shown to play a crucial role in the development of AP in both human and mouse models of AP. Specifically, activation of the NLRP3 inflammasome in macrophages leads to an increased proinflammatory response, which contributes to systemic inflammatory response syndrome (SIRS) [11]. Furthermore, uric acid crystals have been shown to activate the inflammasome, posing the hypothesis that cholesterol or bilirubinate crystals could induce NLRP3 activation in the pancreas [12].

Based on this, using human papilla biopsies and in vitro/in vivo experiments, our study aims to address the gap of how sludge and microlithiasis can trigger AP in the absence of duct obstruction [11, 13].

## 2 Methods

### 2.1 Human Specimens

**FIGURE 1:**
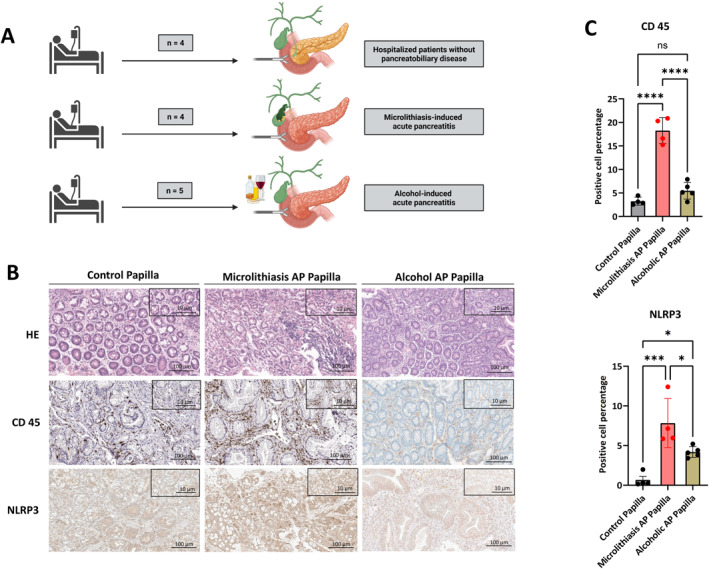
Collection and Quantification of Human Papillary Samples. (A) Human papillary biopsies were prospectively collected from patients with microlithiasis‐induced acute pancreatitis (*n* = 4), alcohol‐induced acute pancreatitis (*n* = 5), and control subjects without pancreatobiliary disease (*n* = 4). (B) Light microscope images of hematoxylin and eosin (H&E), CD45, and NLRP3 immunohistochemistry (IHC) staining (Brightfield: magnification, 100X; scale bars, 10 μm, 100 μm). (C) QuPath quantification for CD45 and NLRP3‐positive cells from the entire tissue. Data points and mean ± SEM are shown. *p ≤ 0.05, **p ≤ 0.01, ***p ≤ 0.001, ****p < 0.0001.

The study was conducted in accordance with the Declaration of Helsinki. Human papillary biopsies were collected under ethics approvals 19–277 and 25–0804 (Ethics Committee of LMU University, Germany) and PCN/0022/KB1/139/19 and 37/2025/B (Ethics Committee of the Medical University of Silesia/Rzeszów, Poland). All participants provided written informed consent prior to inclusion, with the consent process explicitly addressing potential risks associated with papillary biopsy, including bleeding, edema, and post‐procedural pancreatitis. Biopsies were performed by experienced endoscopists following standard safety protocols. No cases of post‐procedural pancreatitis or other major procedure‐related complications were observed in either the study or control groups. Papillary biopsies were obtained from patients with endosonographically confirmed microlithiasis‐induced AP (*n* = 4), alcohol‐induced AP (*n* = 5), and PDAC (*n* = 5), and from individuals undergoing gastroduodenoscopy without pancreatobiliary disease (*n* = 4) (Figure 1A). Of note, no adverse or severe adverse events were recorded due to biopsies of the papilla. In each patient with acute pancreatitis, other potential etiologies were excluded, and biliary microlithiasis was confirmed endosonographically in the common bile duct. Biopsies were embedded in paraffin blocks and stained with HE, anti‐CD45 (Monoclonal Mouse Anti‐Human, concentration 1:100, Leukocyte Common Antigen, Clone 2B11 + PD7/26, CAT#M0701, Dako, Denmark), and anti‐NLRP3 (Human/Mouse NLRP3/NALP3 Antibody, concentration 1:100, R&D; CAT# MAB7578; Minneapolis, MN; United States) antibodies.

### 2.2 Isolation, Culture and Experiments With Bone Marrow‐Derived Macrophages

For bone marrow‐derived macrophages (BMDMs), tibia and fibula bones were collected after cervical dislocation of mice. After removing muscle and fat, bones were cut at both ends, bone marrow was flushed with ice‐cold PBS and cells were cultured in RPMI 1640 media supplemented with MCSF (20 ng/mL). For the differentiation of macrophages from M0 to M1 macrophages, INF‐ (20 ng/mL) and LPS (100 ng/mL) were used as stimulators (change of medium on days 1 and 3, experiments carried out on day 5). Macrophages (M0 + M1) were incubated with the cholesterol monohydrate and calcium bilirubinate crystals in different concentrations (LPS + ATP (5 mM), 125 μg/mL +/− LPS, 375 μg/mL +/− LPS). LPS was used as a priming stimulus (signal 1) to induce transcription of pro‐IL‐1β, consistent with the canonical two‐signal model required for NLRP3 inflammasome activation in macrophages [14, 15]. After incubation of the crystals with macrophages, the supernatant was collected at 0, 3, 6, and 24h, centrifuged at 4°C with 10.000 rpm for 5 min, and stored immediately at −80°C for further analysis. For immunofluorescence staining, macrophages were cultured in 8‐well chamber slide, primed with LPS/ATP(5 mM) stimulation, and after 24 h treatment with the cholesterol monohydrate and calcium bilirubinate crystals (125 μg/mL +/− LPS, 375 μg/mL +/− LPS), the supernatant was removed. The cells were gently washed with PBS to remove crystals and fixed with 4% paraformaldehyde at room temperature for 20 min. Following fixation, cells were washed with PBS. Cells were then permeabilized with 0.1% Tween‐20/PBS for 15 min and blocked for 1h in 5% donkey serum, 1% goat serum and incubated with primary antibodies: NLRP3 (concentration 1:400, R&D; CAT#MAB7578; Minneapolis, MN; United States), ASC (concentration 1:500 CAT#sc‐514414 Santa Cruz Biotechnology Inc. Dallas, Texas, United States) overnight at 4°C. Afterward, the cells were washed in PBS and incubated with corresponding secondary antibodies tagged with Cy3 or FITC for 1h at room temperature. Afterwards cells were washed in PBS and counterstained with DAPI to stain the nucleus for 10 min and kept at 4°C for 20 min in the dark. The final fluorescence pictures were captured using a Carl‐Zeiss Microscope (Axio imager.m2) and ZEN 3.4.91.0 software. Details on the histological analysis of mouse papillae are reported in the Supporting Information S1.

### 2.3 Biochemical Measurements

In cell supernatant, IL‐1β cytokine concentration was determined using an enzyme‐linked immunosorbent assay (ELISA) kit (Biolegend ELISAMax Deluxe Set Mouse IL‐1β (Cat # 432615)) as per the manufacturer's protocol. Immunoblotting for caspase‐1 (pro and active form) was performed using macrophage homogenates as described previously [11]. For the mouse model, blood serum levels of lipase, amylase, alkaline phosphatase (ALP), glutamate pyruvate transaminase (GPT) and lactate dehydrogenase (LDH) were analyzed using the Cobas 8000 system as per the manufacturers protocol under strict quality control at the Department of Clinical Chemistry, Technical University Munich, Germany.

### 2.4 Development of a Microlithiasis‐Induced Mouse Model for Biliary Pancreatitis

**FIGURE 2:**
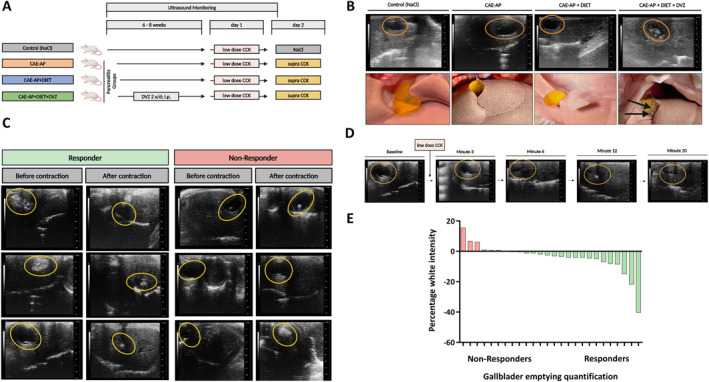
Experimental design and ultrasonography of mice gallbladder. (A) Flow chart of the experimental design, depicting the four experimental groups (control (NaCl), CAE‐AP, CAE‐AP + DIET and CAE‐AP + DIET + DVZ), weekly ultrasound, low (40 ng/kg) and high (50 μg/kg) dose CCK and DVZ (1 mg/kg) injection. (B) Visualization of the gallbladder on ultrasound (upper row) and macroscopically (lower row) with evidence of microlithiasis in the CAE‐AP + DIET + DVZ group (see arrow). (C) Ultrasound images of responder versus non‐responder mice gallbladder before and after low‐dose CCK‐induced gallbladder contraction (8x). (D) Ultrasound image of gallbladder contraction after low‐dose CCK injection. (E) Gallbladder emptying quantification in terms of increase or decrease in white intensity (Microliths) in responder versus non‐responder experimental groups. The results are shown from two independent experiments.

All animal experiments were carried out in compliance with REMARK guidelines, after ethical approval from the Regierung von Mecklenburg‐Vorpommern and the Regierung von Oberbayern (proposal number: TVA_ROB_55.2–2532.Vet_02‐17–85). Female Balb/c mice aged 6–8 weeks were obtained from Charles River Inc. and housed in a standard pathogen‐free environment. Following 1 week of acclimatization, the mice were randomly divided into four treatment groups: (1) control (NaCl), (2) CAE‐AP (=Caerulein‐induced acute pancreatitis), (3) CAE‐AP + (high fat) DIET and (4) CAE‐AP + (high fat) DIET + devazepide (DVZ). Groups 3 and 4 were given a lithogenic high‐fat diet (lithogenic diet: Altromin C 1080; 1% cholesterol and 0.5% bile acids) orally over a period of 6–8 weeks instead of the mice's normal diet. Group 4 received additional morning and evening *i.p.* Injections of the CCK1 receptor antagonist devazepide ( 1 mg/kg) over the same period. The increased lithogenicity of bile caused by the high‐fat diet, combined with the devazepide‐induced CCK‐mediated gallbladder hypomotility, led to microlithiasis formation in Group 4, which was monitored weekly by gallbladder ultrasound [16]. Once Group 4 developed microlithiasis in all mice, eight low‐dose CCK *i.p.* Injections (40 ng/kg) with one‐hour intervals were administered on the penultimate day of the experiment (day 1) to induce gallbladder contraction and microlithiasis migration into the duodenum. For methodological comparability, low‐dose CCK was also administered at the same dose and frequency in the groups without microlithiasis (groups 1 and 2). On the last day of the experiment (day 2), pancreatitis was induced in all groups by administering supramaximal caerulein *i.p.* Injections (50 μg/kg) eight times with one‐hour intervals and the mice were sacrificed 1 hour after the last injection (Figure 2A). Further details on the ultrasound procedure are available in the supplement.

## 3 Histology, Immunohistochemistry and Immunofluorescence Staining

Paraffin‐embedded tissue sections (3 μm) were deparaffinized, rehydrated and stained using hematoxylin and eosin (H&E) [17]. For immunohistochemistry staining, after deparaffinization and rehydration, tissue sections were boiled in 1X citrate buffer (pH 6.0) to retrieve epitopes, blocked for endogenous peroxidase activity with 3% H_2_O_2_/PBS, and then incubated with 1% BSA/PBS. Sections were incubated with different primary antibodies overnight at 4°C. For the mouse tissue sections, the following primary antibodies were used: CD45 (D3F8Q Rabbit mAb concentration 1:100, Cell Signaling; CAT# 70257; Danvers, Massachusetts, United States), F4/80 (concentration 1:100, Cell Signaling; CAT# D2S9R; Danvers, Massachusetts, United States), CD206 (concentration 1:100 R&D; CAT# AF2535; Minneapolis, MN; United States Followed by secondary antibody incubation, Anti‐Rabbit, ready to use; CAT# K4003; Agilent Technologies Inc. (Dako Envision) and Anti‐goat IGg, concentration 1:300; CAT# 705–035‐003 Jackson immuno research Lab Inc. Visualization of the staining was performed using the DAB Substrate Chromogen System (CAT# SK‐4100, Vector Laboratories Inc. CA, United States) with hematoxylin as the counterstain. Whole slide scans using (3D HISTECH KFT Pannoramic MIDI‐042109, Budapest, Hungary) of the pancreas were performed and positive cell detection was performed by using QuPath software. All pathological and immunohistochemical sections were independently evaluated by two observers blinded to the experimental groups. Scoring was performed according to standardized criteria for inflammation, edema, necrosis, and cellular injury, and blinding was maintained throughout to minimize observer bias.

### 3.1 Statistical Analysis

Data are presented as mean ± standard error of the mean (SEM). To assess the statistical significance, non‐parametric Mann‐Whitney test, the non‐parametric Kruskal‐Wallis test followed by Dunn's multiple comparison test, and One‐way Analysis of Variance (ANOVA) with Tukey's multiple comparisons were performed using GraphPad Prism 9 software (GraphPad Software Inc.). Results with a *p*‐value below 0.05 were considered statistically significant, with levels of significance denoted as follows: *p < 0.05, **p < 0.01, ***p < 0.001, and ****p < 0.0001.

## 4 Results

### 4.1 Increased NLRP3 Activation in Papilla Biopsies of Microlithiasis‐AP Patients

HE staining of the papilla vateri of the AP patients showed altered histological architecture evident by edema, damage to epithelial cell linings and immune cell infiltration. We also observed a significant increase in total immune cell infiltration by CD45 staining in papillae from microlithiasis‐induced acute pancreatitis patients compared with alcohol‐induced acute pancreatitis and control subjects. Additionally, we detected a significantly higher expression of NLRP3 in microlithiasis AP papillae (Figure 1B + C). NLRP3 expression was highest in the PDAC cohort, whereas CD45 infiltration was greatest in the microlithiasis‐induced AP cohort compared with all other groups, including PDAC (Suppl. Fig. 1). In summary, the examined microlithiasis‐induced AP papillae showed signs of papillitis, whereas no corresponding inflammatory phenotype was detectable in the papillae from alcohol‐induced acute pancreatitis and control subjects.

### 4.2 Cholesterol Monohydrate and Calcium Bilirubinate Crystals Induce NLRP3 Inflammasome Activation in BMDMs

**FIGURE 3:**
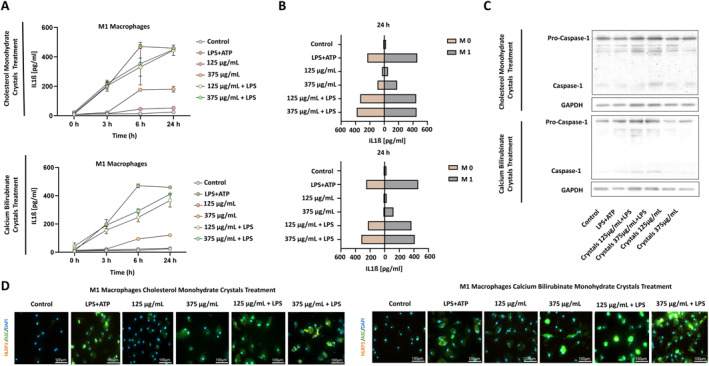
Activation of the NLRP3 inflammasome in vitro. (A) IL1 beta expression in supernatant at different time points upon cholesterol monohydrate and calcium bilirubinate crystal stimulation of M1 macrophages. (B) Comparison of cholesterol monohydrate and calcium bilirubinate crystal‐induced IL1 beta expression at 24 h in M0 (non‐differentiated) and M1 (differentiated, pro‐inflammatory) macrophages, measured by ELISA. (C) Immunoblot analysis showing procaspase 1 and caspase one expression in M1 macrophages after 24 h of cholesterol monohydrate and calcium bilirubinate crystals stimulation. (D) Light microscopy images of M1 macrophages stimulated with cholesterol monohydrate and calcium bilirubinate crystals in different treatment groups, indicating merged immunofluorescence staining of NLRP3 (orange), ASC (green), and nuclei (DAPI, blue) (Brightfield; magnification, x100; scale bar, 100 μm). Data points and mean ± SEM from three independent experiments are shown.

M0 and M1 differentiated macrophages from BMDMs were incubated with different concentrations [ranging from 125 to 375 μg/mL] of cholesterol monohydrate and calcium bilirubinate crystals for 3, 6 and 24 h. ELISA results from the supernatant of M0 macrophages treated with crystals revealed a dose dependent significantly increased IL‐1β expression, which was further increased by adding LPS priming. A similar pattern of IL‐1β expression was observed in M1 macrophage crystal‐treated supernatants (Figure 3A). Intriguingly, M1macrophages showed significantly higher IL‐1β levels in all treatment groups compared to M0 macrophages, depicting the proinflammatory characteristics of M1 macrophages. Both cholesterol and calcium bilirubinate crystals triggered IL‐1β expression in dose dependent manner; however, IL‐1β levels were higher in the cholesterol‐treated supernatants than in the calcium bilirubinate‐treated group (Figure 3B). We assessed the expression of procaspase‐1 and caspase‐1 as downstream targets of NLRP3. Macrophages treated with crystals 375 μg/mL + LPS, and crystals 125 μg/mL + LPS groups showed significantly higher expression of procaspase‐1 and caspase‐1 (Figure 3C, Figure S5 and S6). To assess the NLRP3 and ASC expression in M1 macrophages, we performed immunofluorescence staining. We treated M1 macrophages with cholesterol monohydrate and calcium bilirubinate crystals for 24h. We observed the highest expression of both NLRP3 and ASC in 375 μg/mL crystal + LPS group. The high expression of ASC indicates that the highly activated proinflammatory pathway later leads to the assembly and activation of the NLRP3 inflammasome resulting in IL‐1β expression and secretion. In calcium bilirubinate crystal‐treated M1 macrophages, the NLRP3 and ASC recruitment followed the same pattern as in cholesterol monohydrate crystal‐treated M1 macrophages (Figure 3D).

### 4.3 Ultrasound‐Based Microlithiasis Monitoring in Mice

To validate the human papilla and in vitro findings, we developed a novel microlithiasis‐based mouse model using a lithogenic diet combined with the CCK antagonist Devazepide. Ultrasound examination after 6–8 weeks of lithogenic diet and reduced gallbladder motility revealed the highest microlithiasis burden in the CAE‐AP + DIET + DVZ group, whereas no microliths were detected in the other groups (Figure 2B). Ultrasound monitoring before and after low‐dose CCK injections demonstrated successful microlith discharge in most mice. In some animals, however, the microlith load increased, likely due to gallbladder contraction compacting the stones within the gallbladder (Figure 2C–E).

### 4.4 Biochemical Changes Across Experimental Groups in Biliary Pancreatitis

**FIGURE 4:**
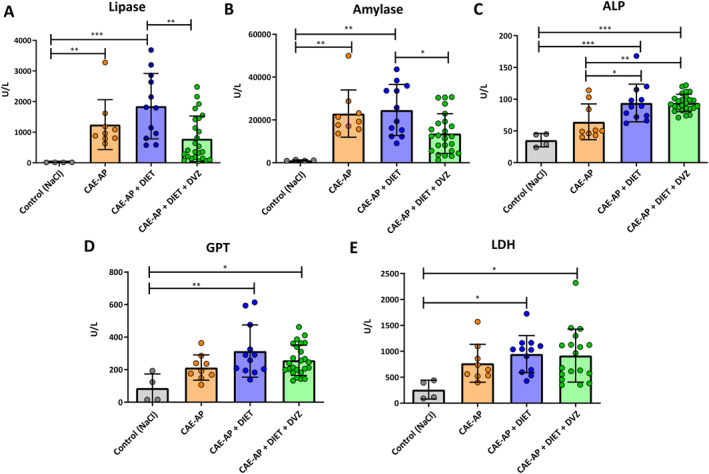
Biochemical profile of the microlithiasismouse model. Mice serum concentrations in (U/L) of (A) Lipase (B) Amylase (C) ALP (D) GPT (E) LDH in different experimental groups. Data points and mean ± SEM from two independent experiments are shown. *p ≤ 0.05, **p ≤ 0.01, ***p ≤ 0.001, ****p < 0.0001.

Lipase was notably higher in the CAE‐AP + DIET group compared with both the control group and the CAE‐AP + DIET + DVZ group. A significant difference in lipase levels was also noted between the control group and the CAE‐AP group (Figure 4A). Similarly, amylase levels were significantly elevated in both the CAE‐AP and CAE‐AP + DIET groups compared with the control group. Furthermore, amylase levels were higher in the CAE‐AP + DIET group than in the CAE‐AP + DIET + DVZ group (Figure 4B). Alkaline phosphatase (ALP) levels were significantly higher in the CAE‐AP + DIET and CAE‐AP + DIET + DVZ groups compared to the control group. Additionally, ALP levels were significantly higher in the CAE‐AP + DIET and CAE‐AP + DIET + DVZ groups than in the CAE‐AP group (Figure 4C). Glutamate pyruvate alanine aminotransferase (GPT), was significantly increased in the CAE‐AP + DIET and CAE‐AP + DIET + DVZ groups compared with the control group (Figure 4D). Furthermore, lactate dehydrogenase (LDH) was significantly higher in the CAE‐AP + DIET and CAE‐AP + DIET + DVZ groups compared to the control group (Figure 4E).

### 4.5 Assessment of Tissue Damage and Immune Cell Infiltration in Biliary Pancreatitis Models

**FIGURE 5:**
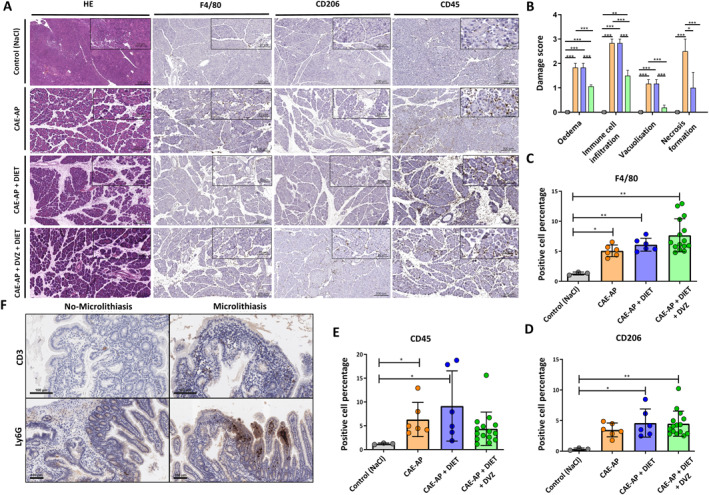
Immunohistochemistry and Quantification in Mice. (A) Histology images depicting hematoxylin and eosin (H&E) staining, along with F4/80 and CD206 staining across various experimental groups(scale bar, 10 and 100 μm). (B) Quantification of the H&E damage score, reflecting histological alterations such as edema, immune cell infiltration, vacuolization, and necrosis among the experimental groups. Positive immune cell detection and QuPath quantification for (C) F4/80, (D) CD206, and (E) CD45 from the entire tissue. Data points and mean ± SEM from two independent experiments are presented. *p ≤ 0.05, **p ≤ 0.01, ***p ≤ 0.001, ****p < 0.0001. (F) Immunostaining for CD3 and Ly6G to compare inflammation of the mouse papillae between microlithiasis mice and non‐microlithiasis mice (scale bar 100 μm).

To investigate intact tissue architecture, H&E staining showed that in comparison to the control group, all other experimental groups showed histopathological signs of inflammation in the pancreas (Figure 5A). The CAE‐AP and CAE‐AP + DIET groups showed the most significant histological changes. H&E damage scoring, based on edema, immune cell infiltration, vacuolisation and necrosis, showed edema and immune cell infiltration in the CAE‐AP, CAE‐AP + DIET and CAE‐AP + DIET + DVZ groups, with necrosis observed only in the CAE‐AP and CAE‐AP + DIET groups (Figure 5B, Figure S7). Quantification showed the highest levels of (F4/80+) macrophages in the CAE‐AP + DIET + DVZ group, followed by the CAE‐AP + DIET and CAE‐AP groups, relative to the control group (Figure 5C). The CAE‐AP + DIET + DVZ group also exhibited the highest anti‐inflammatory M2 (CD206+) macrophage count, followed by the CAE‐AP + DIET and CAE‐AP groups compared to the control group (Figure 5D). CD45 staining indicated significantly higher immune cell infiltration in the CAE‐AP and CAE‐AP + DIET groups compared to the control group (Figure 5E). Mild to moderate peripapillary infiltration with CD3+ cells was present in 2/6 microlithiasis mice and 0/4 non‐microlithiasis mice as well as a moderate infiltration of peripapillary tissue and apical regions of the adjacent duodenal villi with Ly6G + cells (neutrophils) in 2/6 microlithiasis mice and 1/4 non‐microlithiasis mice, respectively (Figure 5F).

## 5 Discussion

The pathomechanism by which biliary microlithiasis or sludge can lead to AP without mechanical bile duct obstruction has not yet been elucidated. In 2002, the inflammasome was first described as a cytosolic multi‐protein complex expressed in myeloid cells involved in inflammatory processes as part of the innate immune system [18]. Since then, it has been characterised as a relevant proinflammatory signaling pathway in innate immunity. In particular, the role of the NLRP3 inflammasome has been characterized in AP and various other inflammatory diseases [11, 19, 20]. Recently, Sendler et al. have shown that activation of NLRP3 aggravates the course of pancreatitis, and inhibition of the NLRP3 inflammasome attenuates pancreatitis [11]. The activation of the NLRP3 inflammasome is triggered by various agonists, such as pathogen‐associated molecular patterns (PAMPs) and danger‐associated molecular patterns (DAMPs) such as uric acid crystals or bacterial surface components [21, 22, 23, 24]. DAMPs such as ATP, HSP70 or HMGB1, originating from damaged acinar cells, can trigger TLR4‐mediated NLRP3 inflammasome activation and a subsequent increase in proinflammatory cytokines in early stages of AP [25, 26]^,^. We demonstrate that the core constituents of sludge and microlithiasis ‐ cholesterol and calcium bilirubinate crystals ‐ are sufficient to activate the canonical NLRP3 inflammasome in BMDMs following LPS priming. The magnitude of IL‐1β release induced by these crystals was comparable to that reported for urate crystals, a prototypical crystalline inflammasome activator. [27] Together with the markedly increased NLRP3 expression observed in human papillae from microlithiasis‐associated AP patients ‐ compared with alcohol‐induced AP and controls ‐ and the inflammatory changes observed in murine papillae following microlith discharge from the gallbladder, these findings support a mechanistic pathway for pancreatitis driven by biliary microlithiasis.

**FIGURE 6:**
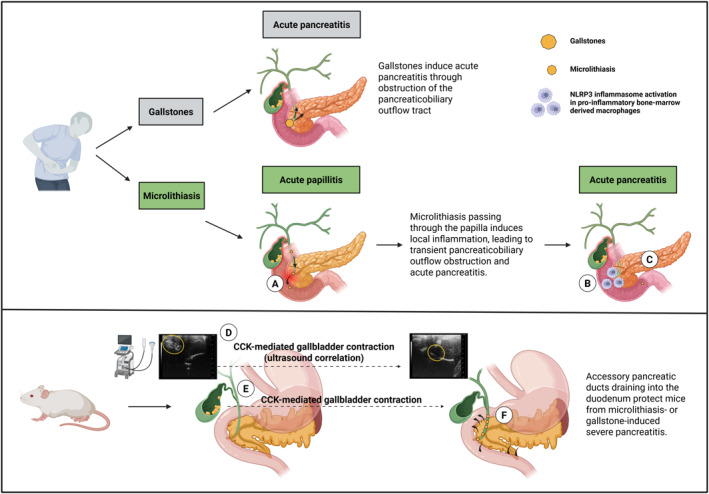
Summary flow chart illustrates the pathophysiological mechanisms of papillitis in humans and mice. (A) Microlithiasis induced localized damage (acute papillitis) at the level of the papilla in humans. (B) Recruitment of pro‐inflammatory M1 macrophages at the level of the papilla in humans. (C) Acute pancreatitis secondary to temporary obstruction of the outflow tract caused by acute papillitis. (D and E) Ultrasonographic correlation image illustrating CCK‐induced gallbladder contraction (gallbladder filled with gallstones or microliths vs. an empty and contracted gallbladder) in mice. (F) Direct and independent drainage of accessory pancreatic ducts into the duodenum in mice.

Biliary crystals, once ejected from the gallbladder, may act as DAMPs by initiating an inflammatory response in the area of the papilla of Vater during their passage into the duodenum, thereby secondarily triggering obstruction through an inflammatory edema (Figure 6). In case of AP triggered by sludge and microlithiasis, this would allow for various treatment options other than cholecystectomy such as endoscopic sphincterotomy or the use of UDCA [28]. For endoscopic sphincterotomy and the associated improved drainage of sludge/microlithiasis into the duodenum without causing further damage to the papilla, there are already qualitative data from studies in non‐operable patients who showed significantly fewer pancreaticobiliary complications in follow‐up [29, 30].The improved drainage of sludge and microlithiasis into the duodenum following endoscopic sphincterotomy might minimise the likelihood of renewed inflammation in the papilla as a trigger for recurrent pancreatitis.

To validate our human biopsy and in vitro findings, we developed a mouse model that mimics human biliary pancreatitis. Different studies have tried to establish models of biliary pancreatitis using retrograde bile salt infusion, sodium taurocholate ductal injection, and other surgical manipulations. However, these approaches are technically demanding and only partially mimic the pathophysiological process observed in humans. In particular, biliary sludge/microlithiasis and its pathophysiological role in the context of AP have not been mimicked by the existing models [10, 31, 32]. In the present study, using a lithogenic diet and devazapide, we developed for the first time a novel biliary pancreatitis mouse model by generating microlithiasis as an endogenous trigger of AP. However, we only noticed significant changes in serum lipase, amylase, ALP, GPT, and LDH levels after caerulein‐induced AP was triggered and not without caerulein. Although we did not include a control group of mice fed a high‐fat diet ± DVZ without pancreatitis, previous studies indicate that a high‐fat diet alone can modestly elevate liver enzymes such as ALT and AST, reflecting mild hepatocellular stress. [33] The mild severity of biliary pancreatitis in our mouse model likely reflects the distinctive anatomical and physiological features of the murine biliary–pancreatic system, which limit ductal obstruction under low‐dose CCK stimulation. The presence of multiple small accessory pancreatic ducts that drain independently into the duodenum, bypassing the papilla of Vater, prevents even substantial microlith discharge from generating sufficient pancreaticobiliary outflow obstruction to exacerbate acute pancreatitis [34]. In addition to the murine anatomical features, the CCK_1_ receptor antagonist devazepide appears to have exerted an additional protective effect on caerulein‐induced pancreatitis. Despite considering devazepide's half‐life, biochemical and histological analyses revealed a milder AP phenotype compared with caerulein‐induced AP combined with a high‐fat diet [35, 36].

Several limitations are inherent to this initial description of the mechanism and should be addressed in future studies. The sample size for human papillary specimens was small, which may limit the generalizability of the findings. This reflects the practical challenge of identifying patients with EUS‐defined biliary microlithiasis in the common bile duct and performing papillary biopsies at the appropriate time, given the high likelihood of stone migration into the duodenum. Nevertheless, to our knowledge, this is the first report presenting a histological analysis of the papilla from patients with microlithiasis‐induced AP alongside alcohol‐induced AP. A papilla biobank is currently being established at LMU University Hospital, which aims to provide a more mechanistic understanding of pancreatitis through the analysis of larger patient cohorts. While our human biopsy data demonstrate clear morphological and immunohistochemical evidence of papillitis, it remains uncertain whether these changes represent a primary pathogenic driver or a secondary consequence of pancreatitis (Figure 1, Figure S4). However, the striking difference between microlithiasis associated and alcohol induced pancreatitis, when it comes to inflammation of the papilla might hint to causality, even not experimentally proven.

Although the study succeeded for the first time in integrating microlithiasis into an AP mouse model and successfully monitoring the discharge of microlithiasis from the gallbladder using sonography, other animals such as sheep or cats are better suited to mimicking partial or complete occlusive microlithiasis duct blockages due to their human‐like pancreatobiliary anatomy. Therefore, microlithiasis alone did not induce pancreatitis in our mouse model (see Suppl. Fig. 2 and 3). Endoscopic interventions are also conceivable due to the size of the animals. Unlike in our mouse model, in such a model, a more severe microlithiasis‐induced AP phenotype would also allow for phenotype rescue experiments using NLRP3 inhibitors [29, 30].

## 6 Conclusion

In summary, based on human, in vitro, and in vivo data, we describe for the first time a potential mechanism by which microlithiasis can trigger acute pancreatitis, even in the absence of biliary obstruction. Biliary crystals activate the NLRP3 inflammasome in bone marrow–derived macrophages, inducing localized inflammation – papillitis ‐ at the papilla of Vater following crystal migration into the duodenum. This focal inflammatory response may act as an initial trigger for the onset of acute pancreatitis.

## Author Contributions

conceptualization: S.S., M.Ż., U.M.M., J.M.. methodology: S.S., M.A., P.A., S.O., L.K., R.S., C.S., L.F., O.B., A.S., C.S., M.R., K.S., I.R., M.Ż, U.M.M., J.M. formal analysis: S.S., M.A., P.A., S.O., G.B., I.R., U.M.M, J.M. data curation: S.S., M.A., P.A, U.M.M. supervision: U.M.M, I.R., M.M.L, J.M. writing ‐ original draft: S.S., M.A., P.A., U.M.M, J.M. writing – review and editing: All.

## Funding

S.S. is funded by the Deutsche Forschungsgemeinschaft (DFG, German Research Foundation) ‐ 413,635,475 ‐ and the LMU Munich Clinician Scientist Program (MCSP). M.Z. is supported by the United European Gastroenterology Research Fellowship. P.A. is funded by the Deutsche Forschungsgemeinschaft (DFG, German Research Foundation) – BE‐63951 and the EU‐IMI2 TransBioLine consortium 821,283. G.B. is supported by the LMU Munich Advanced Clinical Scientist Program (MCSP).

## Conflicts of Interest

The authors declare no conflicts of interest.

## Supporting information


Supporting Information S1

**Figure S1:** Collection and Quantification of Human Papillary Samples. (A) Human papillary biopsies were prospectively collected from patients with microlithiasis‐induced acute pancreatitis (n = 4), alcohol‐induced acute pancreatitis (*n* = 5), PDAC patients (*n* = 5) and control subjects without pancreatobiliary disease (*n* = 4). (B) Light microscope images of hematoxylin and eosin (H&E), CD45, and NLRP3 immunohistochemistry (IHC) staining (Brightfield: magnification, x100; scale bars, 10 μm, 100 μm). (C) QuPath quantification for CD45 and NLRP3‐positive cells from the entire tissue. Data points and mean ± SEM are shown. *p ≤  0.05, **p  ≤  0.01, ***p  ≤  0.001, ****p  <  0.0001.

**Figure S2:** Serum lipase concentrations were measured in mice following low‐dose caerulein (40 ng/kg) treatment. Across all experimental groups and time points, lipase levels remained within a narrow range. In control (NaCl) mice, only minor temporal fluctuations were observed, without evidence of enzymatic elevation. Similarly, low‐dose CAE‐induced pancreatitis, either alone or in combination with diet or diet plus DVZ, did not result in a sustained or marked increase in serum lipase at 3, 7, 11, or 24 hours. Although a transient increase was detected at 3 hours in the low‐dose CAE‐AP group, lipase values rapidly returned to baseline thereafter. Overall, these findings indicate that low‐dose caerulein, with or without dietary or pharmacological modulation, does not elicit a robust systemic lipase response under the applied experimental conditions.

**Figure S3:** LDH release was measured using the Pierce LDH Cytotoxicity Assay Kit (Thermo Fisher Scientific, Cat. no. 88953) according to the manufacturer’s protocol. HPDE cells were seeded in 96‐well plates at a density of 1 × 10⁴ cells per well and allowed to adhere for 24 h. Cells were then treated as indicated for 24 h. Culture supernatants were collected, and absorbance was measured at 490 and 680 nm using a microplate reader. LDH activity was determined by subtracting the background absorbance at 680 nm from the absorbance at 490 nm. Cytotoxicity was calculated relative to low and high controls using the following formula: (experimental LDH release − low control)/(high control − low control) × 100. All experiments were performed in triplicate and repeated independently three times. Triton X‐100 was used as the positive control and induced maximal cytotoxicity. In contrast, exposure to cholesterol and bilirubin crystals at both 125 and 375 µg/mL resulted in significantly lower LDH release compared with the positive control, indicating substantially reduced membrane damage. Among the crystal‐treated groups, cytotoxicity remained moderate and did not approach the levels observed with Triton X‐100, with a slight dose‐dependent decrease observed for bilirubin crystals. These data demonstrate that, under the applied conditions, cholesterol and bilirubin crystals induce only limited cytotoxicity compared with Triton X‐100–mediated cell lysis (Figure S2).

**Figure S4:** Macroscopic Images of the Papilla.

**Figure S5:** Stability of GAPDH expression following calcium bilirubinate crystal treatment. GAPDH protein expression was assessed by densitometric analysis of Western blot bands and normalized to the control group. Experimental conditions included Control, LPS + ATP, and calcium bilirubinate crystals (125 and 375 µg/mL). Data are presented as mean ± SEM from three independent biological experiments (*n* = 3). Statistical analysis was performed using one‐way ANOVA followed by Tukey’s multiple comparisons test. No statistically significant differences in GAPDH expression were observed among groups (*p* > 0.05), confirming stable GAPDH expression under these experimental conditions.

**Figure S6:** Stability of GAPDH expression following cholesterol monohydrate crystal treatment. GAPDH protein expression was assessed by densitometric analysis of Western blot bands and normalized to the control group. Experimental conditions included Control, LPS + ATP, and cholesterol monohydrate crystals (125 and 375 µg/mL). Data are presented as mean ± SEM from three independent biological experiments (*n* = 3). Statistical analysis was performed using one‐way ANOVA followed by Tukey’s multiple comparisons test. No statistically significant differences in GAPDH expression were observed among groups (*p* > 0.05), confirming stable GAPDH expression under these experimental conditions.

**Figure S7:** Total pancreatic injury score. The total pancreatic injury score was calculated for each animal by summing the individual histopathological subscores for oedema, immune cell infiltration, vacuolisation, and necrosis (each graded on a 0–4 scale). Data are presented as mean ± SEM (NaCl, *n* = 4; CAE‐AP, *n* = 6; DIET, *n* = 6; DVZ, *n* = 16) from two independent experiments. Salysis was performed using one‐way ANOVA followed by Tukey’s post hoc multiple comparisons test. *p ≤ 0.05, **p ≤ 0.01, ***p ≤ 0.001, ***p < 0.0001.

## Data Availability

The data that support the findings of this study are available from the corresponding author upon reasonable request.
