# Supplementary figures and images for: NLRP3 Inflammasome Activation‐Induced Acute Papillitis as a Trigger of Acute Pancreatitis ‐ A Novel Mechanism of Microlithiasis‐Induced Acute Pancreatitis
Source: United European Gastroenterol J. 2026 Mar 14;14(2):e70198. doi: 10.1002/ueg2.70198 (PMC13098079; doi:10.1002/ueg2.70198)

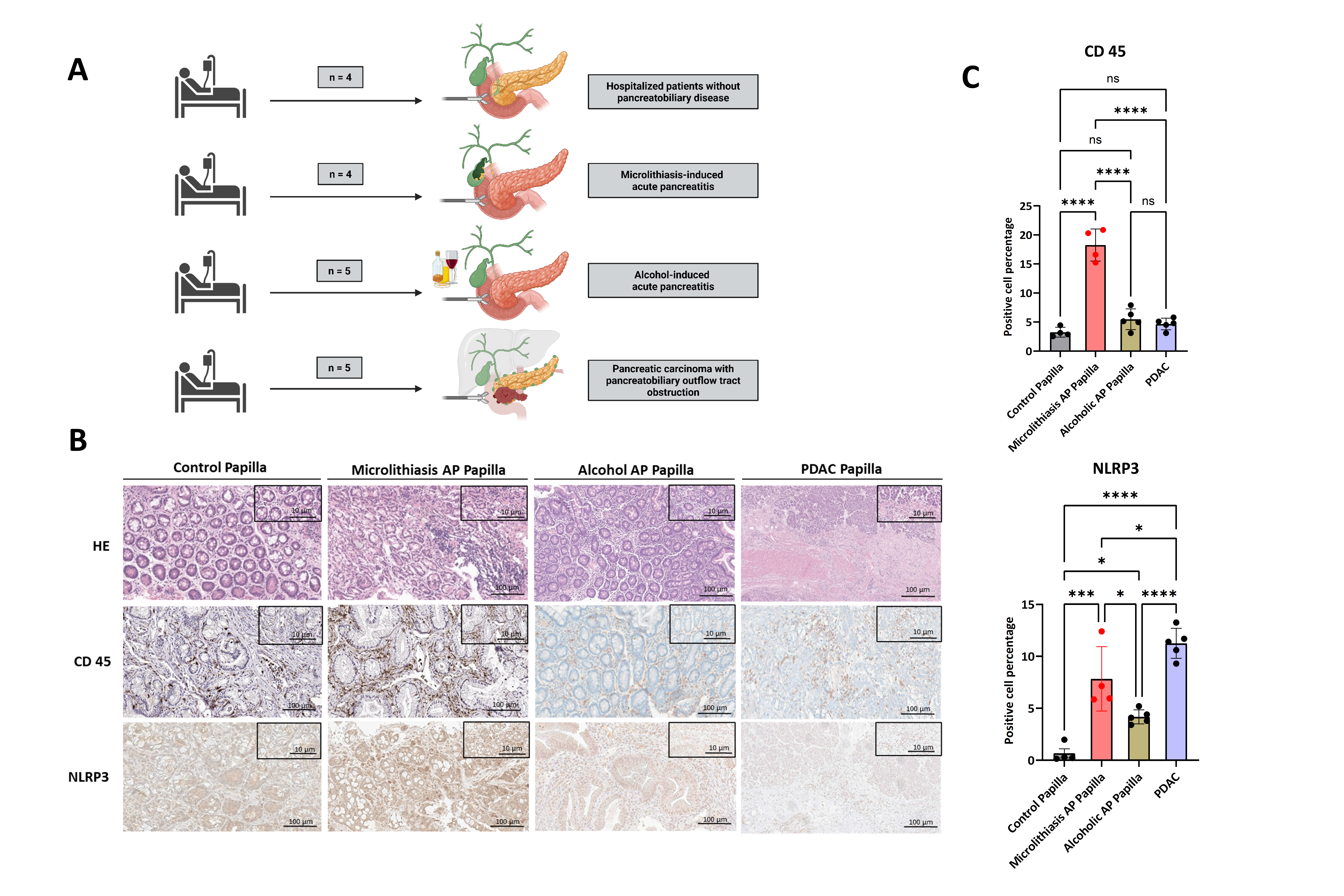

Supplement: Supplementary file 2 — Figure S1: Collection and Quantification of Human Papillary Samples. (A) Human papillary biopsies were prospectively collected from patients with microlithiasis‐induced acute pancreatitis (n = 4), alcohol‐induced acute pancreatitis (n = 5), PDAC patients (n = 5) and control subjects without pancreatobiliary disease (n = 4). (B) Light microscope images of hematoxylin and eosin (H&E), CD45, and NLRP3 immunohistochemistry (IHC) staining (Brightfield: magnification, x100; scale bars, 10 μm, 100 μm). (C) QuPath quantification for CD45 and NLRP3‐positive cells from the entire tissue. Data points and mean ± SEM are shown. *p ≤ 0.05, **p ≤ 0.01, ***p ≤ 0.001, ****p < 0.0001. [file UEG2-14-e70198-s002.png]

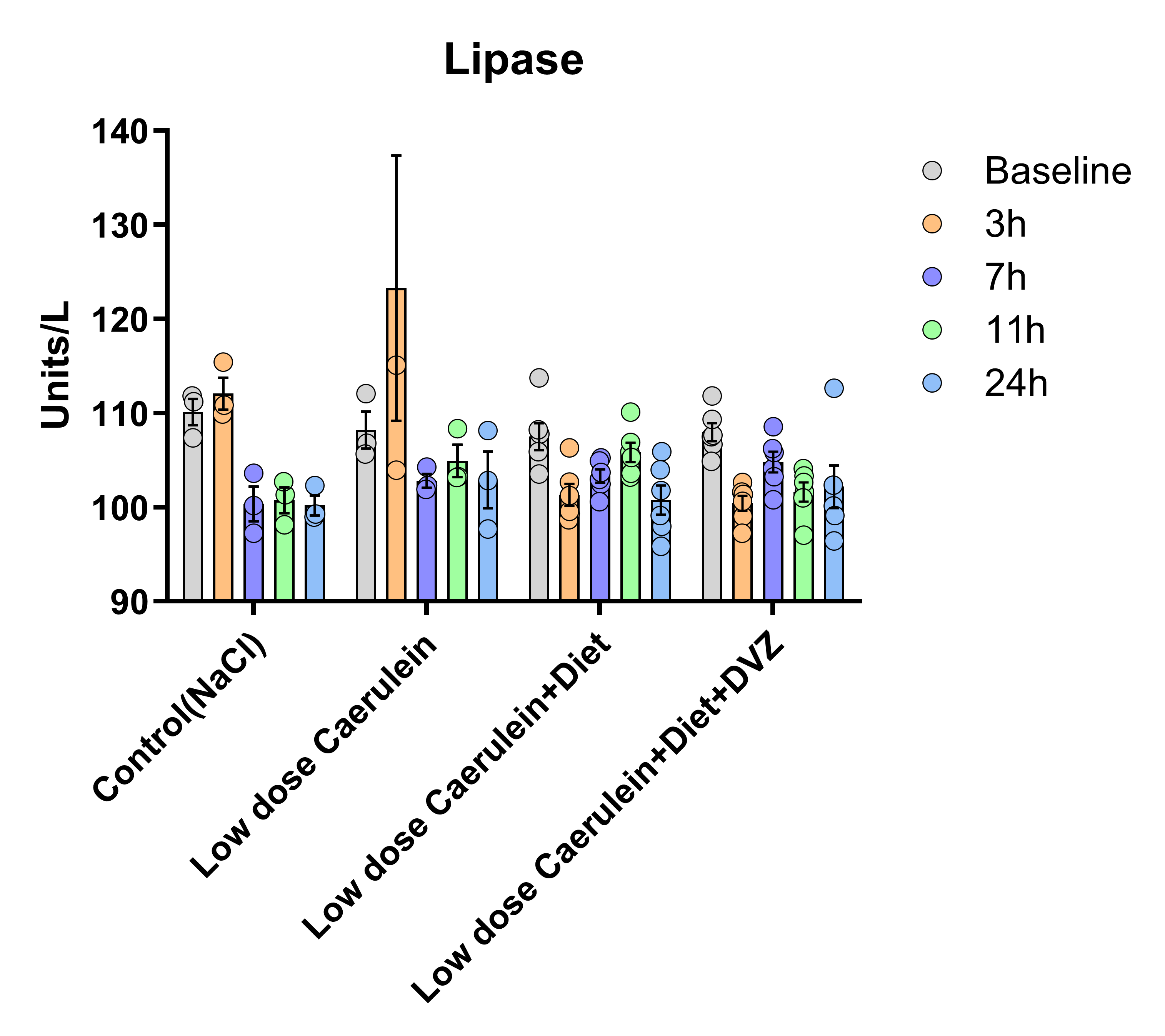

Supplement: Supplementary file 3 — Figure S2: Serum lipase concentrations were measured in mice following low‐dose caerulein (40 ng/kg) treatment. Across all experimental groups and time points, lipase levels remained within a narrow range. In control (NaCl) mice, only minor temporal fluctuations were observed, without evidence of enzymatic elevation. Similarly, low‐dose CAE‐induced pancreatitis, either alone or in combination with diet or diet plus DVZ, did not result in a sustained or marked increase in serum lipase at 3, 7, 11, or 24 hours. Although a transient increase was detected at 3 hours in the low‐dose CAE‐AP group, lipase values rapidly returned to baseline thereafter. Overall, these findings indicate that low‐dose caerulein, with or without dietary or pharmacological modulation, does not elicit a robust systemic lipase response under the applied experimental conditions. [file UEG2-14-e70198-s004.png]

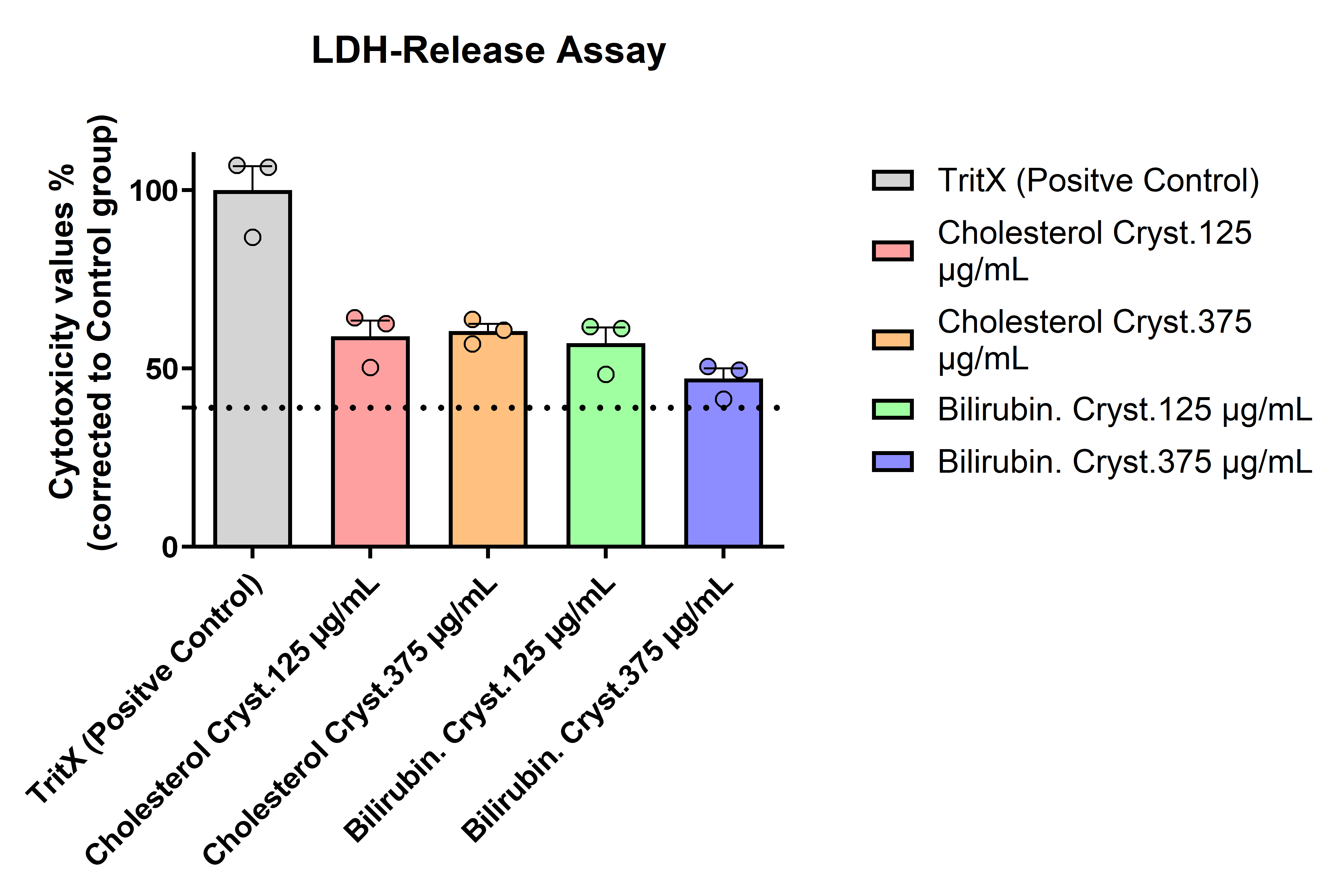

Supplement: Supplementary file 4 — Figure S3: LDH release was measured using the Pierce LDH Cytotoxicity Assay Kit (Thermo Fisher Scientific, Cat. no. 88953) according to the manufacturer’s protocol. HPDE cells were seeded in 96‐well plates at a density of 1 × 10⁴ cells per well and allowed to adhere for 24 h. Cells were then treated as indicated for 24 h. Culture supernatants were collected, and absorbance was measured at 490 and 680 nm using a microplate reader. LDH activity was determined by subtracting the background absorbance at 680 nm from the absorbance at 490 nm. Cytotoxicity was calculated relative to low and high controls using the following formula: (experimental LDH release − low control)/(high control − low control) × 100. All experiments were performed in triplicate and repeated independently three times. Triton X‐100 was used as the positive control and induced maximal cytotoxicity. In contrast, exposure to cholesterol and bilirubin crystals at both 125 and 375 µg/mL resulted in significantly lower LDH release compared with the positive control, indicating substantially reduced membrane damage. Among the crystal‐treated groups, cytotoxicity remained moderate and did not approach the levels observed with Triton X‐100, with a slight dose‐dependent decrease observed for bilirubin crystals. These data demonstrate that, under the applied conditions, cholesterol and bilirubin crystals induce only limited cytotoxicity compared with Triton X‐100–mediated cell lysis (Figure S2). [file UEG2-14-e70198-s006.png]

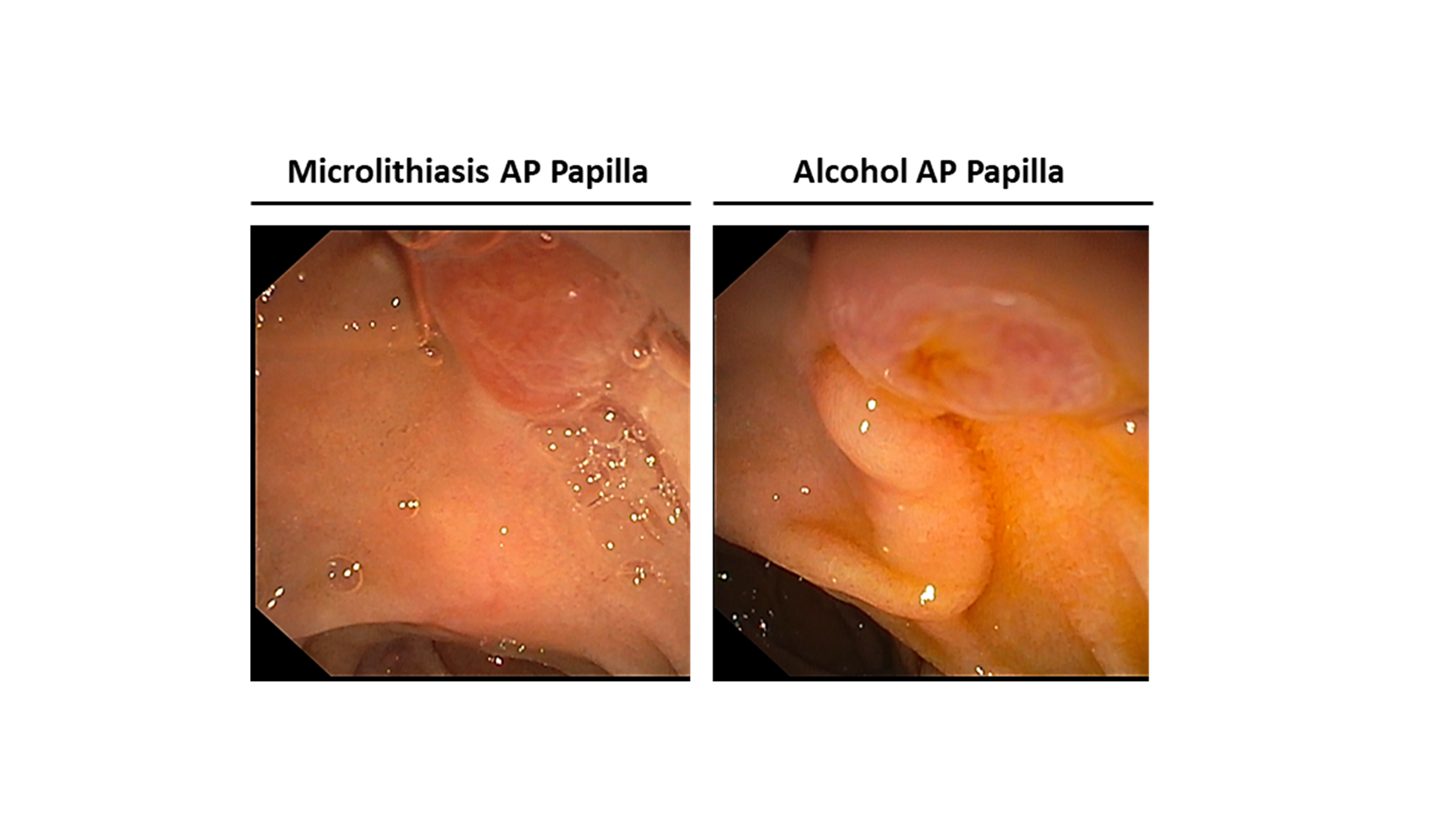

Supplement: Supplementary file 5 — Figure S4: Macroscopic Images of the Papilla. [file UEG2-14-e70198-s005.png]

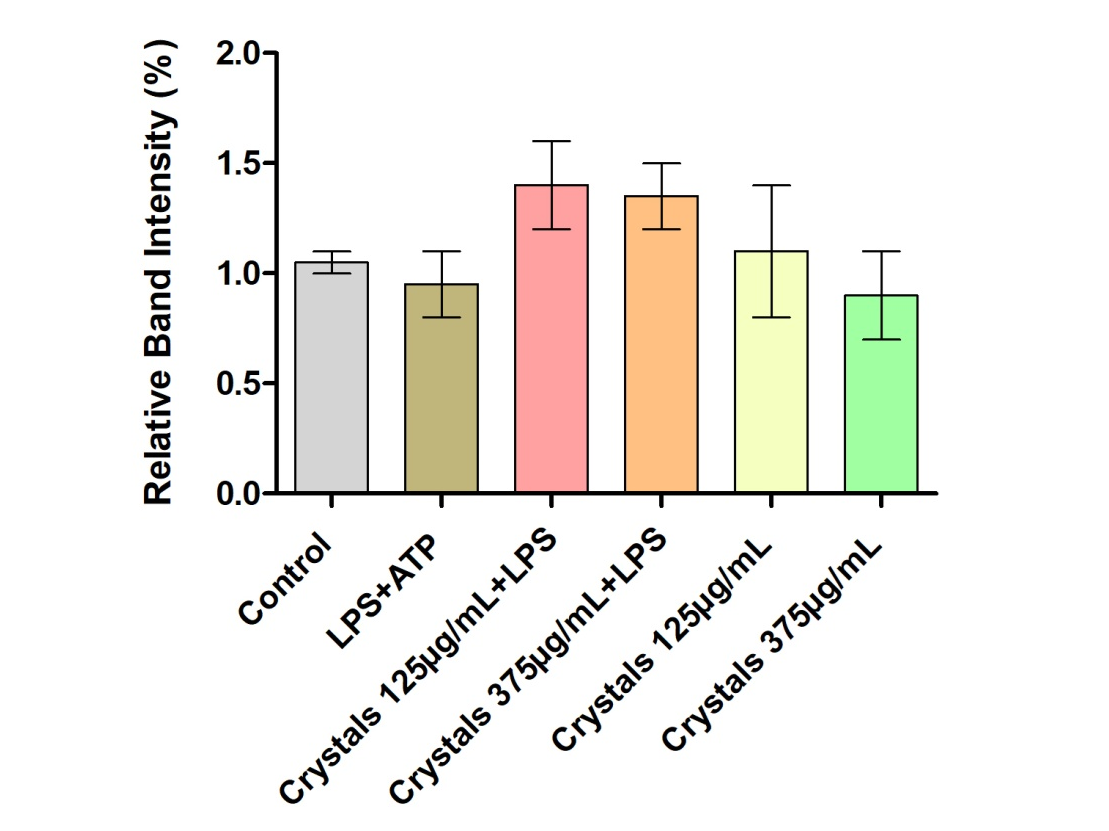

Supplement: Supplementary file 6 — Figure S5: Stability of GAPDH expression following calcium bilirubinate crystal treatment. GAPDH protein expression was assessed by densitometric analysis of Western blot bands and normalized to the control group. Experimental conditions included Control, LPS + ATP, and calcium bilirubinate crystals (125 and 375 µg/mL). Data are presented as mean ± SEM from three independent biological experiments (n = 3). Statistical analysis was performed using one‐way ANOVA followed by Tukey’s multiple comparisons test. No statistically significant differences in GAPDH expression were observed among groups (p > 0.05), confirming stable GAPDH expression under these experimental conditions. [file UEG2-14-e70198-s008.png]

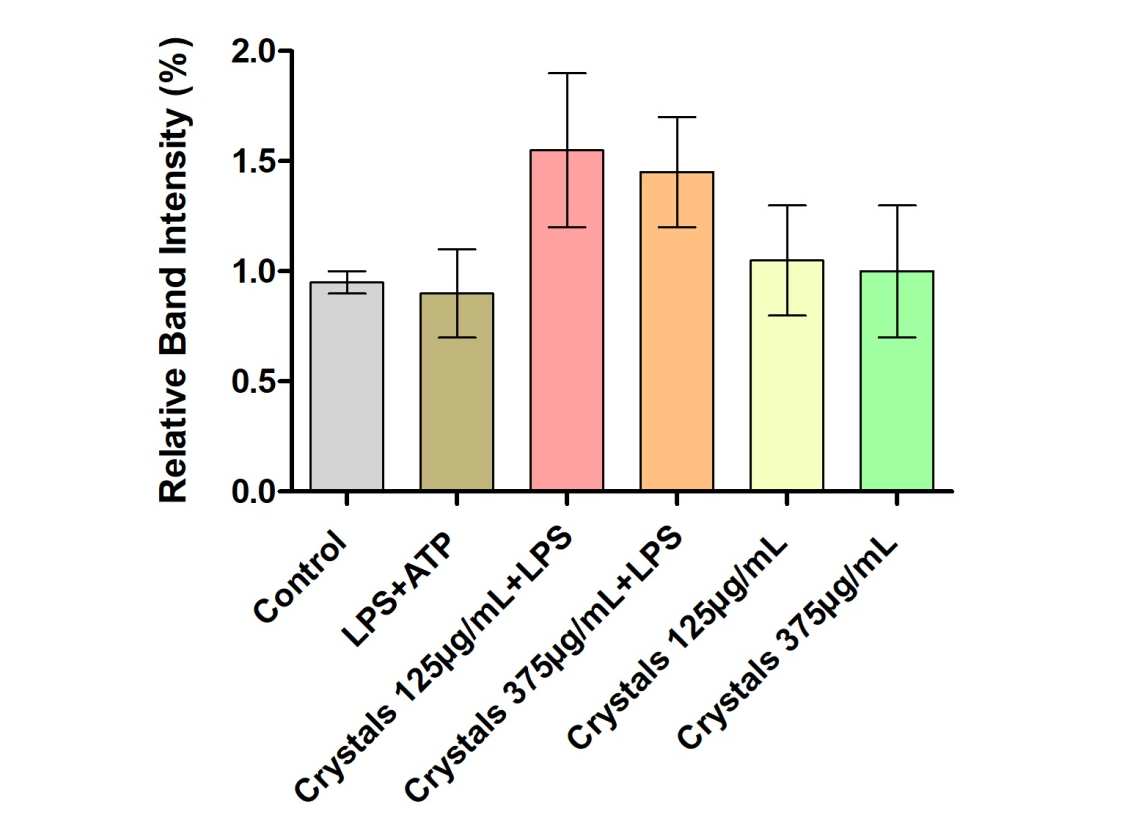

Supplement: Supplementary file 7 — Figure S6: Stability of GAPDH expression following cholesterol monohydrate crystal treatment. GAPDH protein expression was assessed by densitometric analysis of Western blot bands and normalized to the control group. Experimental conditions included Control, LPS + ATP, and cholesterol monohydrate crystals (125 and 375 µg/mL). Data are presented as mean ± SEM from three independent biological experiments (n = 3). Statistical analysis was performed using one‐way ANOVA followed by Tukey’s multiple comparisons test. No statistically significant differences in GAPDH expression were observed among groups (p > 0.05), confirming stable GAPDH expression under these experimental conditions. [file UEG2-14-e70198-s007.png]

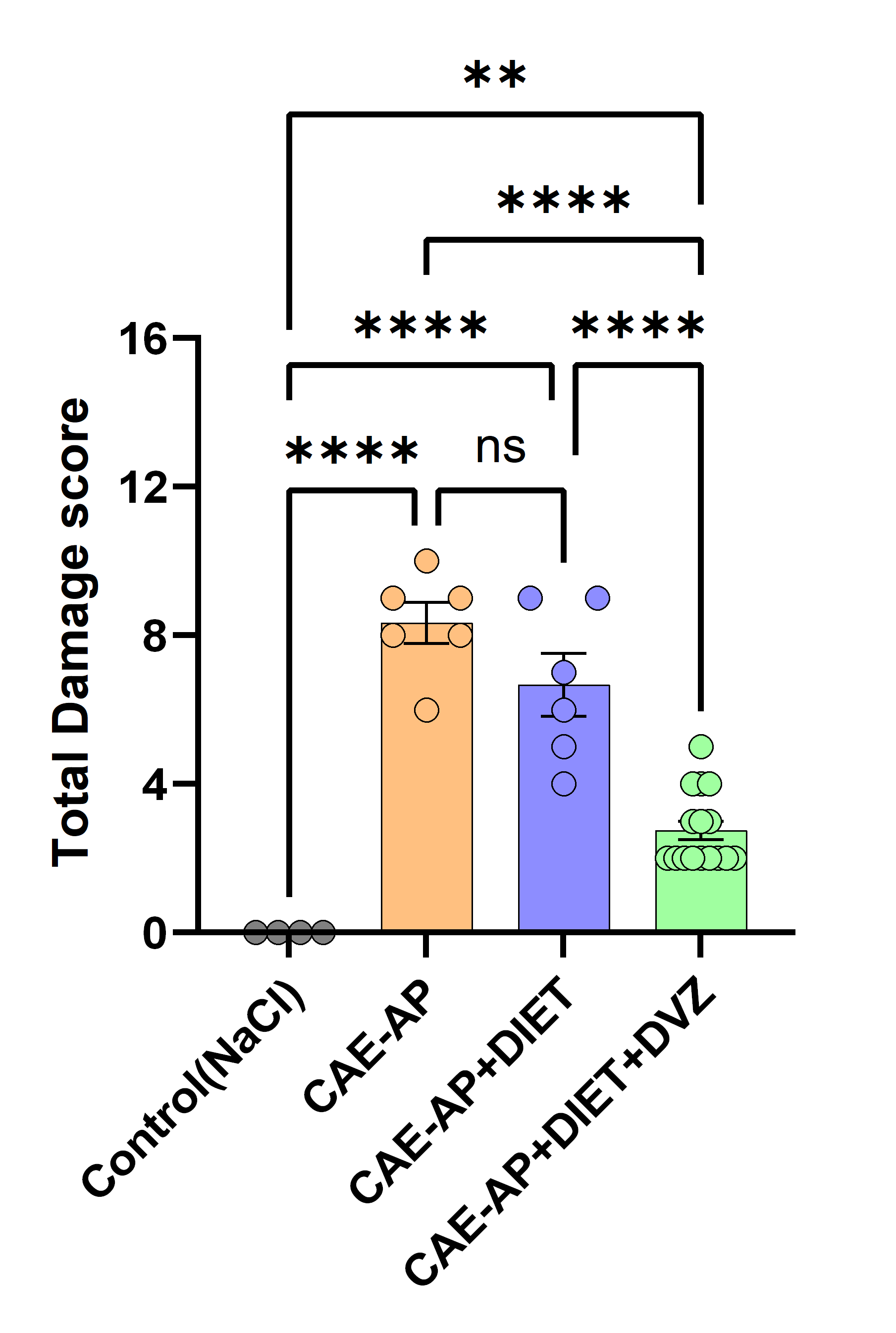

Supplement: Supplementary file 8 — Figure S7: Total pancreatic injury score. The total pancreatic injury score was calculated for each animal by summing the individual histopathological subscores for oedema, immune cell infiltration, vacuolisation, and necrosis (each graded on a 0–4 scale). Data are presented as mean ± SEM (NaCl, n = 4; CAE‐AP, n = 6; DIET, n = 6; DVZ, n = 16) from two independent experiments. Salysis was performed using one‐way ANOVA followed by Tukey’s post hoc multiple comparisons test. *p ≤ 0.05, **p ≤ 0.01, ***p ≤ 0.001, ***p < 0.0001. [file UEG2-14-e70198-s003.png]
